# Artificial intelligence-generated and algorithmically personalized music interventions and their effects on brain function, behavior, and health: a critical review

**DOI:** 10.3389/fnhum.2026.1967909

**Published:** 2026-10-23

**Authors:** Jingting Jin

**Affiliations:** School of Education, Jilin International Studies University, Changchun, Jilin, China

**Keywords:** affective computing, artificial intelligence, closed-loop neurofeedback, EEG, generative music, music intervention, neural entrainment, personalization

## Abstract

This critical review synthesizes empirical studies of algorithmically personalized and artificial-intelligence-generated music interventions reporting neurophysiological, neuroimaging, or objective behavioral outcomes. Literature was identified through structured searches of PubMed, Scopus, Google Scholar, IEEE Xplore, and the ACM Digital Library through 12 August 2026, with CNKI and Wanfang searched on 22 September 2026, supplemented by citation tracking. Twenty-one eligible studies (at least 1,005 participants) were identified. Five things the term artificial intelligence covers in this literature are separated: algorithmic selection from a corpus, rule-based sonification, parametric modulation of pre-existing material, machine-learning generative composition, and closed-loop delivery contingent on the listener’s own signal. Only three studies used a machine-learning generative model, so most of the evidence concerns algorithmic personalization of existing material. Studies of closed-loop neural systems reported the most consistent neurophysiological signal, and music-based alpha neurofeedback produced higher normalized alpha power than yoked random feedback. In an open-loop design, personalized EEG-derived music was reported to attenuate anxiety induction where self-selected preferred music and silence did not, with enhanced prefrontal theta oscillations as the associated correlate. Eight studies carried a comparator capable of isolating an algorithmic component from music exposure, and all eight favored the algorithmic condition. These eight address different causal questions, use different designs, and point to no single mechanism, so their results are best read as preliminary and hypothesis-generating. Evidence quality was very low throughout. The median reported sample size was 22, no study blinded participants or measured expectancy, and no specific finding has been independently replicated. GRADE certainty was very low for every outcome domain, covering EEG oscillatory power, sleep architecture, autonomic regulation, epileptiform activity, and cognitive, behavioral and affective outcomes. Neurophysiological change is reported more often than behavioral change, and behavioral change more often than clinical benefit. The evidence supports continued investigation and does not support clinical deployment. Eight reporting and design requirements for future trials are proposed.

## 1 Introduction

Music occupies an unusual position among non-pharmacological interventions. It is culturally ubiquitous, inexpensive to deliver, largely free of adverse effects, and engages a distributed network of auditory, limbic, prefrontal, motor, and reward structures rather than any single circuit. Music listening recruits tightly coupled auditory–motor circuitry (Zatorre et al., 2007; Chen et al., 2008; Bengtsson et al., 2009) and elicits striatal dopamine release during peak emotional experience (Salimpoor et al., 2011). Coordinate-based meta-analysis of music-evoked emotion has consistently implicated amygdalar, hippocampal, and ventral striatal involvement alongside auditory cortical processing (Koelsch, 2020). Music-based rehabilitation has correspondingly been associated with both structural and functional neuroplastic change across clinical populations (Särkämö et al., 2014; Blasi et al., 2025). These properties have supported a substantial clinical evidence base spanning dementia care (Lu et al., 2025), stroke rehabilitation, where daily music listening improved verbal memory and focused attention in a landmark randomized trial (Särkämö et al., 2008) and rhythmic auditory stimulation reliably improves post-stroke gait (Yoo and Kim, 2016; Mainka et al., 2018; Gonzalez-Hoelling et al., 2021), as well as sleep disturbance and affective disorder (Zhao et al., 2024).

That evidence base rests on a common and rarely examined assumption: that the musical stimulus is fixed. In the overwhelming majority of trials participants receive either a standardized researcher-selected programme or a self-selected preferred playlist. In both cases the music is chosen in advance and does not change in response to what the listener’s brain or body is doing while they listen. This design choice has consequences that recur throughout the meta-analytic literature. Reviews of music therapy in dementia, in cognitively normal older adults, and in sleep disturbance converge on the same limitation: substantial and largely unexplained between-study heterogeneity, with intervention parameters (genre, tempo, duration, and degree of individual tailoring) repeatedly nominated as the most plausible unmodelled moderators (Lu et al., 2025; Tang et al., 2025; Zhao et al., 2024). An expert consensus on music-based rehabilitation trials likewise identifies stimulus specification and individualization as central unresolved methodological challenges (Grau-Sánchez et al., 2022). Where reviewers have examined tailoring directly, participant musical preference has emerged as a determinant of cognitive outcome rather than an incidental variable (Rajakumar and Mohan, 2024).

A parallel literature, developing largely outside clinical neuroscience, has been building the technical capacity to relax this assumption. Work in affective computing, anchored by benchmark datasets that pair EEG and peripheral physiology with affect ratings during music-video viewing (Koelstra et al., 2012), has produced systems capable of inferring emotional state from electroencephalographic (EEG) and autonomic signals in near real time, and generative modelling has advanced from rule-based playback control toward systems that compose or continuously modulate musical material conditioned on those inferred states (Dash and Agres, 2024). Musical neurofeedback provides the clearest instantiation of the resulting closed loop: the listener’s ongoing neural activity determines the acoustic output, which in turn shapes subsequent neural activity. This is a specialization of the general closed-loop training principle, whose mechanisms and validation requirements are increasingly well characterized (Sitaram et al., 2017). Early applications demonstrated feasibility for affective outcomes in older adults (Ramirez et al., 2015); subsequent work reported that music-based auditory neurofeedback modulated alpha-band power under randomized crossover conditions (Takabatake et al., 2021). Most recently, personalized music generated directly from participants’ own resting-state EEG was reported to enhance anxiety resistance under induced stress relative to both self-selected preferred music and silence (Yang et al., 2025). Two systematic reviews have begun to map the neurofeedback subset of this design space (Bhavsar et al., 2024; Sayal et al., 2025).

Three features distinguish this emerging class of intervention from conventional music therapy in ways that matter neuroscientifically. First, the adaptation latency, meaning the interval between a change in the listener’s state and a corresponding change in the acoustic stimulus, can be reduced from an entire session to seconds, placing the intervention within the timescale of neural oscillatory dynamics rather than that of clinical review. Rhythmic tonal sequences entrain neural oscillations at exactly this timescale, with measurable consequences for subjective emotion (Ding et al., 2025). Second, the personalization signal may be neural rather than behavioral, making the intervention conditional on brain state in a way that self-report-based tailoring cannot achieve. Third, generative systems can produce endlessly non-repeating material, removing the habituation and over-familiarity that constrain repeated exposure to fixed recordings. Each constitutes a testable mechanistic hypothesis about why algorithmic personalization might outperform fixed music, and each is amenable to neurophysiological measurement.

The evidence bearing on these hypotheses is distributed across literatures that do not routinely cite one another. Clinical trials appear in medical and nursing journals; system descriptions and validation studies appear in engineering venues indexed in IEEE Xplore and the ACM Digital Library; theoretical integration has appeared largely in narrative and mini-review form (Jiao, 2025). Existing reviews are either confined to neurofeedback paradigms (Bhavsar et al., 2024; Sayal et al., 2025) or to binaural-beat entrainment (Ingendoh et al., 2023), or are oriented toward system architecture rather than measured human outcomes. No review has yet appraised the full class of algorithmically personalized music interventions with neurophysiological outcomes as the organizing endpoint. Consequently several questions of direct relevance to intervention design remain unanswered: whether closed-loop personalization produces neural effects distinguishable from open-loop or preference-matched control conditions; which signal modalities and adaptation latencies are associated with stronger effects; and whether reported effects reflect the personalization mechanism itself or non-specific factors including novelty, expectancy, and increased engagement.

Listener characteristics shape the response to any given piece of music, and they bear directly on what personalization is supposed to achieve. Lee-Harris et al. (2018) compared two age groups listening to the same meditative binaural material and to calm classical music. The younger group showed larger self-reported arousal change, and the older group rated the low-arousal classical music as the most comforting. Verma (2021) reported that men scored higher than women on cognitive regulation through music listening. Neurohumoral state is a further candidate moderator. Menstrual cycle phase has been examined as a factor in whether music acts as an anxiolytic or a stressor for female professional musicians (Bazanova, 2003), and it alters mood states (Ghazel et al., 2022). The effects of music on exercise performance (Ghazel et al., 2022) and on judgements of facial attractiveness (Marin et al., 2017) did not differ between phases. Neither study tested whether cycle phase alters the anxiolytic effect of music, so the question remains open. Musical training and preference also moderate neural response, and a validated index of musical sophistication is available for measuring it (Müllensiefen et al., 2014). A literature that proposes to tailor music to the individual therefore needs to report which music, delivered under which conditions, produced an effect in which listeners.

This review addresses that gap. Our objectives are to (i) identify and characterize empirical studies of AI-generated or algorithmically personalized music interventions reporting neurophysiological, neuroimaging, or objective behavioral outcomes in humans; (ii) construct a taxonomy of personalization architectures based on sensing modality, adaptation latency, and generative approach; (iii) synthesize reported neural mechanisms and outcome effects across that taxonomy; (iv) appraise risk of bias and the adequacy of control conditions, with particular attention to whether comparators isolate personalization from music exposure per se; (v) characterize the musical material, the acoustic parameters under algorithmic control, and the listening conditions this literature reports; and (vi) derive methodological and reporting recommendations for the next generation of trials.

## 2 Materials and methods

### 2.1 Review approach

This article is a critical review with a structured search and pre-defined eligibility criteria. It is not a registered systematic review, and no claim of exhaustive coverage is made. Eligibility criteria, the search approach, and a classification taxonomy were fixed in advance, so that studies are selected and compared on explicit and reproducible grounds. Chinese-language eligibility and the delivery-contingency dimension were added after the initial search. The term critical review is used throughout. Several instruments developed for systematic reviews are used here as structured appraisal frameworks and not as the formal procedures they constitute in a registered review. PICOS organizes the eligibility criteria, PRISMA 2020 (Page et al., 2021) organizes the reporting, the signalling questions of RoB 2 and ROBINS-I organize the appraisal of individual studies, GRADE organizes the summary of certainty by outcome domain, and SWiM principles organize the narrative synthesis. Screening was carried out by one reviewer without independent duplicate assessment, which raises the potential for selection bias relative to a registered systematic review.

### 2.2 Definition of artificial intelligence and personalization

The term artificial intelligence covers several distinct procedures in this literature, and treating them as equivalent would obscure what the evidence shows. Four ways of producing the musical material are separated here, together with one property of how that material is delivered.

Algorithmic selection from a corpus means a rule or a trained model chooses existing recordings for the listener. Rule-based sonification means features of a physiological recording are mapped to musical parameters by a fixed transformation, so the output is new material derived from a signal. Parametric modulation means an existing piece is played back while a signal changes its parameters, such as tempo, loudness, or added noise. Machine-learning generative composition means a model trained on musical data composes new material, and it is the only category corresponding to what the phrase AI-composed music usually denotes.

Closed-loop delivery is a property of the delivery and cuts across all four. A system is closed-loop when the acoustic output at each moment depends on the listener’s own neural, autonomic, or movement signal at that moment, so the listener’s response feeds back into the stimulus. A system can therefore be generative without being closed-loop, and closed-loop without being generative.

The distinction carries a theoretical claim. Selection, sonification, and generation can all improve how well the stimulus matches the listener’s state, and any benefit they confer is continuous with the benefit of well-chosen music. Closed-loop contingency is the only property that introduces a mechanism with no counterpart in fixed music, because the recursive coupling between state and stimulus is what neurofeedback accounts of learning rest on (Sitaram et al., 2017). The two accounts make different predictions, and the comparative-effects section below reports the studies bearing on each.

### 2.3 Eligibility criteria

Eligibility was defined using the PICOS framework (Table 1).

**Table 1:** Eligibility criteria (PICOS).

Domain	Inclusion	Exclusion
Population	Human participants, any age, healthy or clinical.	Animal studies; purely computational studies with no human participants.
Intervention	Music intervention in which musical content is generated, composed, selected, sequenced, or continuously modulated by a computational, algorithmic, or machine-learning procedure. Includes generative/AI-composed music, affect-conditioned selection, physiologically adaptive systems, and music-based neurofeedback.	Fixed pre-composed playlists; clinician- or participant-selected music without algorithmic mediation; pure-tone or binaural-beat stimulation without musical structure; music as background ambience.
Comparator	Any comparator, including standardized music, self-selected preferred music, non-adaptive algorithmic music, yoked/random feedback, silence, white noise, treatment as usual, or within-subject baseline.	—
Outcomes	At least one neurophysiological (EEG, MEG, fNIRS, HRV, EDA, cortisol), neuroimaging (fMRI, PET), or objectively measured behavioral/cognitive outcome.	Studies reporting exclusively self-report outcomes with no objective measure.
Study design	RCTs, non-randomized controlled trials, crossover, within-subject experimental, pre–post feasibility studies.	Reviews, editorials, protocols without results, case reports (*n* < 3), descriptive system papers without human outcome data.
Reports	Peer-reviewed journal articles and full conference papers. English and Chinese language. Inception to September 2026.	Abstracts, posters, theses, non-peer-reviewed preprints.

Two eligibility decisions warrant explanation. First, we retained full conference papers indexed in IEEE Xplore and the ACM Digital Library, since a substantial proportion of validated adaptive music systems are first reported in engineering venues and restricting to journal articles would systematically exclude the technical core of the field. Second, we required at least one objective outcome. Self-report instruments dominate the conventional music therapy literature and are highly susceptible to expectancy effects. These effects are elevated here, because participants who are told that music is being generated for them by AI have obvious grounds to expect benefit. Requiring an objective endpoint does not eliminate this confound, but it ensures that every included study contributes at least one measure the participant cannot directly report.

### 2.4 Information sources and search strategy

Literature was identified through structured searches of PubMed/MEDLINE, Scopus, Google Scholar, IEEE Xplore, and the ACM Digital Library through 12 August 2026. In Scopus, a two-stage strategy was used: a broad sensitivity search combining the three concept blocks (5,260 records) and a high-precision search requiring fused personalization–music phrases (e.g., “generative music,” “adaptive music,” “brain-wave music,” “musical neurofeedback”) in combination with outcome terms (338 records), with the sensitivity set screened by relevance ranking as a safety net. The search combined three concept blocks (music intervention, algorithmic personalization, and neurophysiological or behavioral outcome) using controlled vocabulary and free-text terms. Reference lists of included studies and of relevant prior reviews (Bhavsar et al., 2024; Dash and Agres, 2024; Sayal et al., 2025) were hand-searched, and forward citation tracking was performed on all included studies.

Chinese-language publications were made eligible, and two Chinese-language databases, CNKI and Wanfang, were searched on 22 September 2026. The same seven subject searches were run in each database, returning 661 records in CNKI and 1,761 in Wanfang. The Chinese search terms reproduced the three concept blocks used in the English strategy. They covered brain-wave music (脑波音乐), music with neurofeedback (音乐 神经反馈), artificial intelligence with music and intervention (人工智能 音乐 干预), and music with generation and EEG (音乐 生成 脑电). They also covered personalized music with intervention (个性化音乐 干预), music with brain-computer interface (音乐 脑机接口), and algorithmic music with EEG (算法音乐 脑电).

The core PubMed strategy is reproduced below; equivalent term combinations were used in the remaining sources.

(music[MeSH] OR “music therapy”[MeSH] OR music*[tiab] OR musical[tiab] OR soundscape*[tiab] OR “auditory stimulation”[tiab]) AND (“artificial intelligence”[MeSH] OR “machine learning”[MeSH] OR “deep learning”[tiab] OR generative*[tiab] OR algorithm*[tiab] OR personali*[tiab] OR individuali*[tiab] OR tailor*[tiab] OR adaptive[tiab] OR “closed-loop”[tiab] OR neurofeedback[tiab] OR biofeedback[tiab] OR “affective computing”[tiab] OR “brain-computer interface”[tiab] OR “brain-wave music”[tiab]) AND (electroencephalograph*[tiab] OR EEG[tiab] OR magnetoencephalograph*[tiab] OR MEG[tiab] OR fNIRS[tiab] OR “near-infrared”[tiab] OR “functional magnetic resonance”[tiab] OR fMRI[tiab] OR polysomnograph*[tiab] OR “heart rate variability”[tiab] OR electrodermal[tiab] OR cortisol[tiab] OR “neural oscillat*”[tiab] OR entrainment[tiab] OR connectivity[tiab] OR cognit*[tiab] OR “reaction time”[tiab] OR “motor performance”[tiab])

### 2.5 Study selection

Titles and abstracts of retrieved records were screened against the eligibility criteria in Table 1, and full texts of potentially eligible records were assessed. Studies retained after full-text assessment were classified against the taxonomy in Table 2, and their data were extracted into a standardized form. Screening and extraction were carried out by one reviewer, without independent duplicate assessment. Single-reviewer screening carries a higher risk of missed records and of inconsistent application of the criteria than dual independent screening does, so the resulting corpus is a characterized evidence base and not an exhaustive census. The search strategy is reported in full so that it can be replicated and extended.

**Table 2:** Classification dimensions for personalization architecture.

Dimension	Levels	Definition
Sensing modality	None (trait/preference only); behavioral/self-report; autonomic (HRV, EDA, respiration); neural (EEG, fNIRS, fMRI); multimodal	The signal on which personalization is conditioned.
Adaptation latency	Static (fixed after initial personalization); session-level; sub-minute; real-time (<5 s)	Interval between a change in listener state and corresponding change in the stimulus.
Generative method	Algorithmic selection from a corpus; parametric modulation of pre-composed material; rule-based/procedural composition; machine-learning generative model	How musical material is produced or altered.
Delivery contingency	Open-loop; closed-loop on the listener’s own neural, autonomic or movement signal	Whether the acoustic output at each moment depends on the listener’s concurrent state.

### 2.6 Data extraction

A standardized extraction form was used. Extracted variables comprised study characteristics; participant characteristics including musical training history and whether a validated musical-sophistication instrument such as the Gold-MSI (Müllensiefen et al., 2014) was administered; intervention architecture, input signal modality, adaptation latency, generative method, and musical parameters under algorithmic control; comparator nature and whether it controlled for music exposure, novelty, and expectancy; outcome measures and analytic approach; and effect estimates with dispersion. All extracted values were verified against the published source at the time of extraction. Extraction also covered the musical material and its source, any genre, composer, or tempo reported, the emotional character targeted, the acoustic parameters placed under algorithmic control, the listening condition and concurrent activity, and the session dose. These variables are reported in Table 3.

**Table 3:** Musical material, parameters under algorithmic control, and listening conditions in the included studies.

Study	Musical material and source	Parameters under algorithmic control	Targeted state	Listening condition	Session dose
Moens et al. (2014)	Commercial tracks from a tempo-matched pool	Playback tempo and beat phase aligned to footfall	Gait synchronization	Walking and running, in the laboratory, on a treadmill, outdoors and at an exhibition	Not reported
Ramirez et al. (2015)	Pieces preselected by the participant	Loudness from frontal beta/alpha ratio; tempo from frontal alpha asymmetry	Depressive state	Clinic session; concurrent activity not reported	10 sessions over 5 weeks
Huang et al. (2016)	Brainwave music composed from the participant’s baseline resting EEG	Rule-based sonification of EEG features	Orofacial pain	During orthodontic tooth movement	Daily over 1 week
Chiu and Ko (2017)	Music selected by a learned preference model	Selection driven by wearable HRV sensing	Task performance	During task performance	Not reported
Fedotchev (2018)	Music driven by individually identified narrow-band EEG oscillators	Contingent narrow-band oscillator feedback	Occupational stress	Session; concurrent activity not reported	2 sessions
Gao et al. (2020)	Brain-wave music derived from sleep-stage EEG (slow-wave sleep and REM variants)	Pitch, duration, volume and timbre mapped from amplitude, period and average power	Sleep quality	Before bedtime, with sleep recorded overnight	20 min nightly for 6 days
Byrns et al. (2020)	Eight 30-s excerpts of popular songs from the listener’s youth, chosen for slow tempo and low pitch	Selection of the excerpt with the best EEG-derived emotional response, replayed for longer	Cognition and emotional state	Immersive virtual-reality theatre	Single session of about 10 min
Li et al. (2020)	Decompression music selected by spectral classification	Selection by spectral class, delivered as neurofeedback	Sub-threshold depression	Session; concurrent activity not reported	10 min daily for 14 days
Takabatake et al. (2021)	Classical music with superimposed white noise	Noise level inversely coupled to normalized alpha power	Alpha power and cognition	Neurofeedback session; concurrent activity not reported	At least 4 weeks per arm
Miyamoto et al. (2022)	Material generated by the system during the session	Generation conditioned on EEG-predicted emotion, on an iso-principle trajectory	Target emotion induction	Laboratory emotion-induction session	Not reported
Wang et al. (2023)	Brain-wave music converted from gaming-state and eye-open resting-state EEG	Rule-based sonification of EEG features	Fatigue	Session following an experimental fatigue induction	Not reported
Hu et al. (2024)	Music selected in a closed loop	Selection driven by ongoing EEG	Student stress	Neurofeedback session; concurrent activity not reported	5 sessions
Wijethunge et al. (2024)	Melodies generated from EEG-identified preferred song segments	Transformer-based generative model output	Work stress	Laboratory listening; concurrent activity not reported	Not reported
Wu et al. (2025)	AI-generated background music	Valence, arousal and motivational tone set per condition	Learning-related well-being	During numerical and visual memory tasks	Not reported
Xu and Li (2025)	Five-tone material in the Jue mode	Modulation rules connecting HRV and EDA to output not specified	Anxiety	Single session; concurrent activity not reported	Single 20-min session
Yang et al. (2025)	Brain-wave music generated from the participant’s own resting-state EEG	Sonification reported at the level of the power-law principle	Anxiety resistance	During experimental anxiety induction	Not reported
Zhu and Zhang (2025)	Context-aware AI-generated music	Adaptation to road conditions	Spatial memory and navigation	During simulated driving	Not reported
Sakakibara et al. (2025)	Popular songs from a recommendation database spanning the 1920s to 2023	Recommendation updated every 20 s from EEG-decoded nostalgia	Nostalgia, well-being and memory vividness	Laboratory session; concurrent activity not reported	Six songs of 20 s in each condition
Song et al. (2025)	Individualized material delivered from interaction logs and physiological signals	Dynamic adjustment; rules not specified	Stress and positive affect	Everyday use	12 weeks
Hećimović et al. (2026)	Culturally relevant commercial tracks matched to audified healthy-hemisphere EEG	Selection by acoustic match to audified EEG	Epileptiform activity and sleep	All night, during sleep	Nightly for 3 weeks
Zhou et al. (2026)	Four music stimulus types selected by a valence-arousal mapping	Selection by valence and arousal targets	Emotional state in smart-home interaction	Laboratory listening; concurrent activity not reported	Not reported

Studies are ordered by year of publication. No study named a composer or a specific work. HRV, heart rate variability; EDA, electrodermal activity; REM, rapid eye movement; SDS, Self-Rating Depression Scale.

### 2.7 Personalization taxonomy

Because interventions in this field are described using inconsistent terminology across clinical and engineering literatures, we developed an *a priori* classification along three orthogonal dimensions, and later added delivery contingency as a fourth, cross-cutting dimension (Table 2). This permits studies to be compared on functional architecture rather than on the labels their authors use.

### 2.8 Risk of bias and certainty of evidence

Methodological quality was appraised for each study with reference to the signalling questions of Cochrane RoB 2 for randomized designs and ROBINS-I for non-randomized designs, applied as a structured appraisal framework rather than as formally adjudicated tool ratings. Two domains received particular scrutiny. Blinding of participants is generally infeasible in music interventions and further complicated where participants know the music is algorithmically generated; we therefore recorded separately whether outcome assessors and analysts were blinded, and whether the study measured or otherwise addressed participant expectancy. We also recorded whether the comparator isolated personalization from music exposure: that is, whether the control group received music at all, and if so whether it was matched for genre, tempo, novelty, and duration. Studies comparing algorithmically personalized music against silence or no intervention cannot attribute any observed effect to personalization, and this distinction is carried explicitly through the synthesis. Certainty of evidence by outcome domain was appraised using GRADE criteria (Table 4).

**Table 4:** GRADE certainty of evidence by outcome domain.

Outcome domain	Studies (*n*)	Risk of bias	Inconsistency	Indirectness	Imprecision	Certainty
EEG oscillatory power (neurophysiological correlate)	9 (256)	Serious	Serious	Not serious	Serious	Very low
Epileptiform activity (clinical neurophysiology)	1 (19)	Serious	Not assessable	Not serious	Serious	Very low
Sleep architecture (neurophysiological correlate)	2 (52)	Serious	Serious	Not serious	Serious	Very low
Autonomic regulation (neurophysiological correlate)	3 (≥540)	Serious	Not serious	Serious	Serious	Very low
Cognitive and behavioral performance (behavioral)	6 (≥51)	Serious	Serious	Serious	Serious	Very low
Affective symptom change (patient-reported clinical)	6 (185)	Serious	Not serious	Not serious	Serious	Very low

Participant totals sum the single enrolment figures reported, and ≥ marks a row that also includes a study without one. The EEG oscillatory power row includes Li et al. (2020) and Wang et al. (2023). The cognitive and behavioral performance row includes Byrns et al. (2020). The affective symptom change row includes Li et al. (2020). The EEG-derived affect indices reported by Byrns et al. (2020) are not counted in the EEG oscillatory power row. Sakakibara et al. (2025) reports EEG-decoded and self-rated nostalgia and well-being in volunteers without a clinical condition, which fit none of the six domains.

### 2.9 Data synthesis

Substantial clinical and methodological heterogeneity was anticipated. Meta-analysis was considered where two or more studies reported a common outcome using comparable measures and comparators; as reported in Section 3.7, no outcome met this condition, and findings were synthesized narratively following SWiM reporting guidance, structured by the taxonomy in Table 2 and by outcome domain. Pre-specified subgroup analyses examined sensing modality, adaptation latency, and population. Publication bias was to be assessed by funnel plot and Egger’s test where ten or more studies contributed to a single meta-analysis.

## 3 Results

### 3.1 Study selection

The high-precision Scopus search returned 338 records after document-type and language limits. Title and abstract screening excluded 273 records, 65 underwent detailed eligibility assessment, and 14 met all criteria. Three further studies known from citation tracking and the other database searches (Takabatake et al., 2021; Xu and Li, 2025; Zhou et al., 2026) were absent from the Scopus export, confirming the value of the supplementary sources. The CNKI and Wanfang searches returned 2,422 records, and 1,601 remained after 821 duplicates were removed. Theses, patents, newspaper items and research-achievement registrations (1,026 records) were removed by record type. The remaining 575 journal and conference records were screened on title and abstract. Six had already been screened in the English-language search, 536 were excluded on title, and 18 were excluded on abstract. Full texts were sought for 15 records. Six could not be retrieved, nine were assessed, and four met all criteria. Two are Chinese-language studies (Li et al., 2020; Wang et al., 2023), and two are English-language studies indexed in Wanfang (Byrns et al., 2020; Sakakibara et al., 2025). The final corpus is therefore 21 studies. Li et al. (2020) is also indexed in PubMed, where it had fallen outside the original English-language criterion. The most common reasons for exclusion at detailed assessment were the absence of any objective outcome measure and descriptive system papers reporting no human outcome data. Other frequent reasons were individualized music delivered by a therapist or selected by preference without algorithmic mediation, emotion-recognition and classification studies in which music was a stimulus for decoding, and case reports with fewer than three participants. Two patterns dominated the Chinese-language records and were excluded on the same grounds as their English-language equivalents. A large clinical literature applies a fixed commercial alpha-wave recording under the name α脑波音乐. The recording is pre-composed and carries no algorithmic mediation. A large nursing literature applies 个性化音乐, meaning music selected by a nurse or by patient preference, and reports self-report outcomes alone.

### 3.2 Study and participant characteristics

The 21 included studies were published between 2014 and 2026 and originated predominantly in East Asia, with eleven studies from China, three from Japan, and one from Taiwan. The remainder came from Spain, Belgium, Russia, Sri Lanka, Canada, and a Croatian–UK collaboration. Designs comprised four randomized controlled or randomized allocation studies, one single-blind randomized crossover trial, one randomized crossover trial of a closed-loop system, several controlled within-subject experiments, and a tail of small pre–post feasibility and pilot studies. Nineteen studies reported sample sizes. Eighteen reporting a single enrolment figure totalled at least 1,005 participants (range 5–500, median 22), and one multi-experiment study contributed between 10 and 119 participants per experiment. Two conference or engineering-venue reports did not state a sample size in the available record. Populations spanned healthy adults and university students, older adults including older adults with subjective cognitive decline, children with epilepsy, orthodontic patients experiencing procedural pain, patients with depression and with sub-threshold depression, workers and first-responder-adjacent occupational groups, drivers, and recreational runners. Populations were predominantly non-clinical. No study reported using a validated instrument to characterize participants’ musical sophistication.

### 3.3 Personalization architectures

Across the 21 included studies, neural sensing predominated (thirteen studies), with two using autonomic signals, two behavioral or contextual sensing, two multimodal input, and two condition-level generation without individual sensing. Adaptation latency divided the corpus sharply. Ten studies operated in real time, updating the acoustic stimulus continuously from an incoming signal (Moens et al., 2014; Ramirez et al., 2015; Chiu and Ko, 2017; Fedotchev, 2018; Li et al., 2020; Takabatake et al., 2021; Miyamoto et al., 2022; Hu et al., 2024; Xu and Li, 2025; Zhu and Zhang, 2025). One operated at sub-minute latency, updating its song selection from decoded EEG every 20 s (Sakakibara et al., 2025). Eight used static personalization, deriving musical material in advance from the participant’s own physiology or preference profile, or setting it at the condition level, and playing it back without further adaptation (Huang et al., 2016; Byrns et al., 2020; Gao et al., 2020; Wang et al., 2023; Wijethunge et al., 2024; Wu et al., 2025; Yang et al., 2025; Hećimović et al., 2026). Two used session-level adaptation (Song et al., 2025; Zhou et al., 2026). The counts in this paragraph correspond one to one with the studies listed at each level and with the cells of Figure 1.

**Figure 1:**
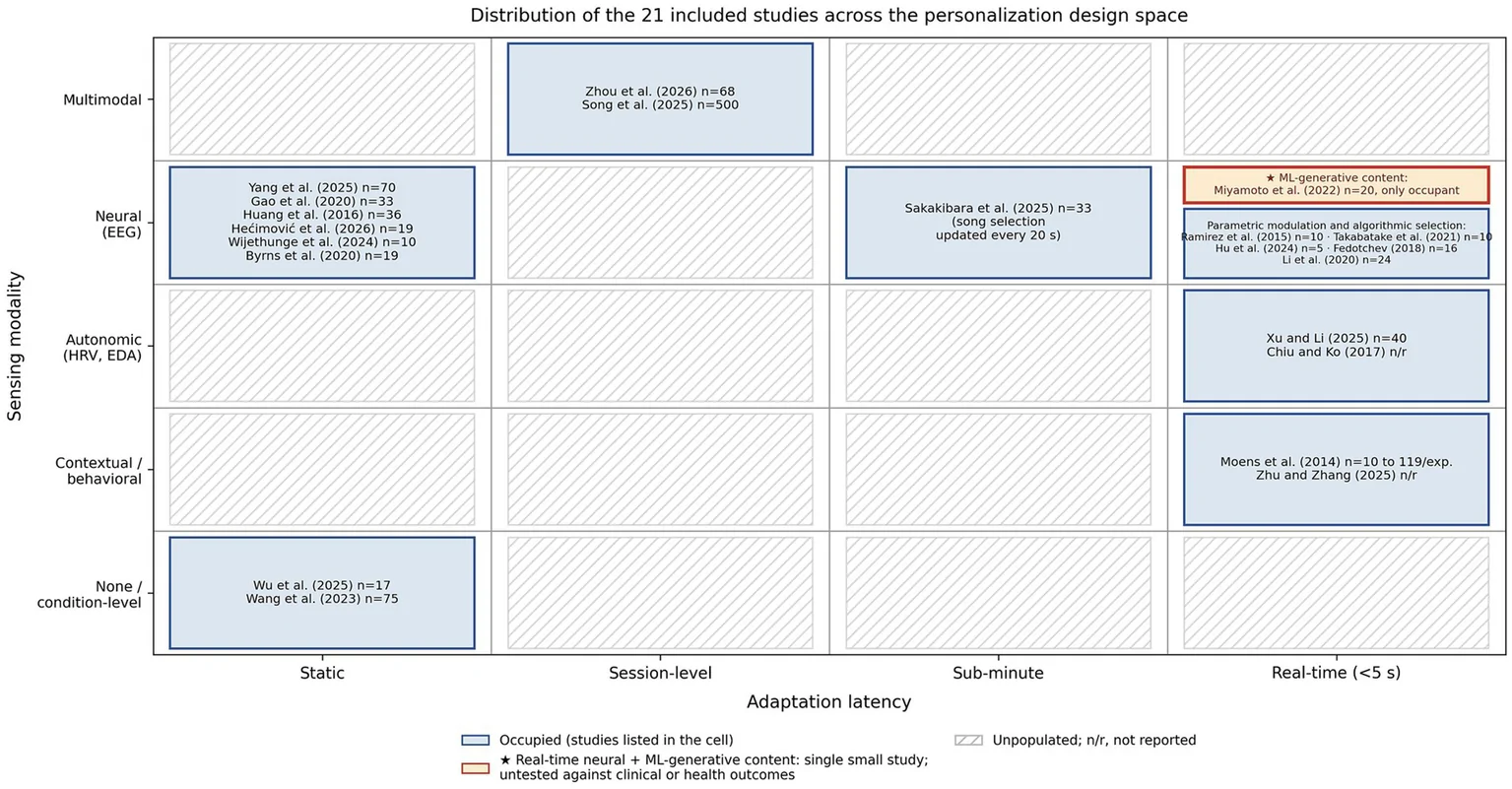
Distribution of the 21 included studies across the sensing-modality and adaptation-latency dimensions of the personalization taxonomy (Table 2). Hatched cells are unpopulated; n/r, sample size not reported. The highlighted portion of the real-time neural cell marks the near-gap discussed in Sections 3.3 and 4.6. Only one small study (Miyamoto et al., 2022) combines real-time neural sensing with machine-learning-generative individualized content, and no study has tested that architecture against a clinical or health outcome.

Classified by how the musical material was produced, eight studies used algorithmic selection from a corpus (Chiu and Ko, 2017; Byrns et al., 2020; Li et al., 2020; Hu et al., 2024; Sakakibara et al., 2025; Song et al., 2025; Hećimović et al., 2026; Zhou et al., 2026), four used rule-based sonification (Huang et al., 2016; Gao et al., 2020; Wang et al., 2023; Yang et al., 2025), five used parametric modulation of pre-existing material (Moens et al., 2014; Ramirez et al., 2015; Fedotchev, 2018; Takabatake et al., 2021; Xu and Li, 2025), and three used a machine-learning generative model (Miyamoto et al., 2022; Wijethunge et al., 2024; Zhu and Zhang, 2025). One further study used AI-generated material whose generative method is not specified in the available record (Wu et al., 2025). Ten studies delivered the output in a closed loop contingent on the listener’s own signal, of which seven used a neural signal (Ramirez et al., 2015; Fedotchev, 2018; Li et al., 2020; Takabatake et al., 2021; Miyamoto et al., 2022; Hu et al., 2024; Sakakibara et al., 2025), two an autonomic signal (Chiu and Ko, 2017; Xu and Li, 2025), and one a movement signal (Moens et al., 2014). Most of the evidence base therefore concerns algorithmic personalization of existing material, and a small part of it concerns music composed by a trained generative model.

The three principal architectures are shown schematically in Figure 2, and the distribution of all 21 studies across the sensing-modality and adaptation-latency dimensions is shown in Figure 1. Six of the static studies personalize the content of the music to the individual’s own neural signature without closing the loop, and two set the material at the condition level with no individual sensing. One used EEG recorded in defined states (Wang et al., 2023), and the other used emotional parameters assigned to each condition (Wu et al., 2025). Most real-time studies close the loop while personalizing by modulating parameters of pre-existing material or by selecting from a corpus. Machine-learning generative models appear in only three studies (Miyamoto et al., 2022; Wijethunge et al., 2024; Zhu and Zhang, 2025), and only one of these, Miyamoto et al. (2022), combines generative content with real-time neural sensing. The theoretical literature identifies this combination as the most promising, and its single occupant is an emotion-induction validation in 20 healthy participants. The architecture remains untested against any clinical or health outcome. The remainder of the corpus used parametric modulation of pre-composed or preselected material, rule-based sonification mapping EEG features to musical parameters, or algorithmic selection from a corpus, despite the prominence of generative models in the parallel engineering literature (Dash and Agres, 2024).

**Figure 2:**
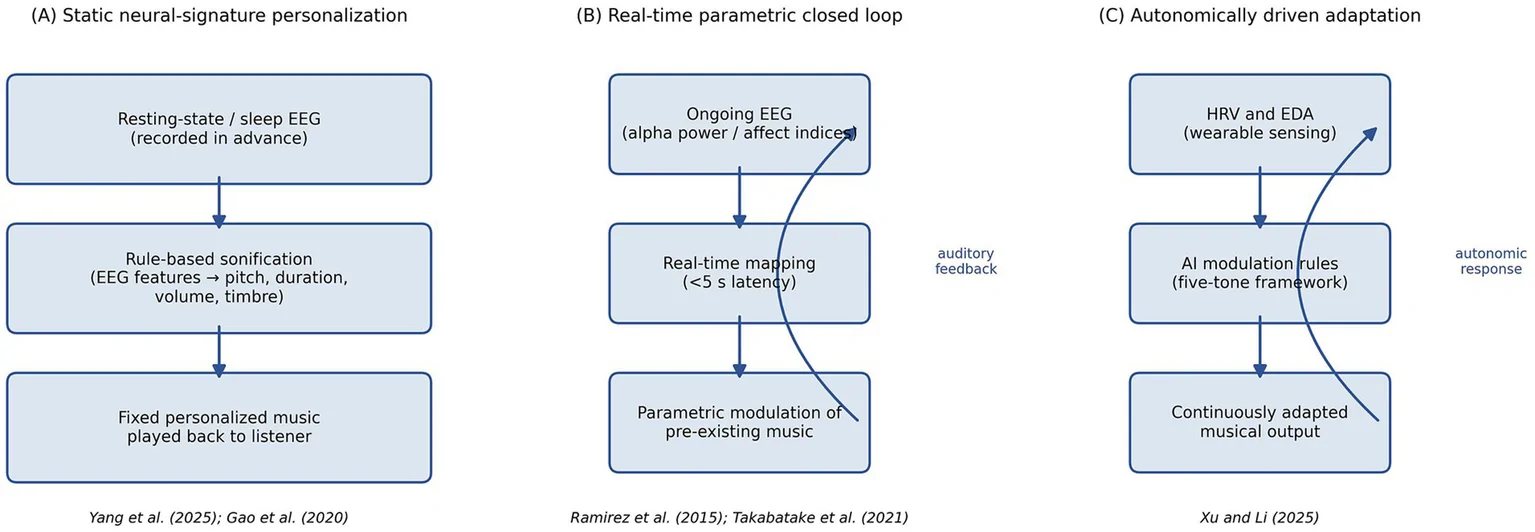
Schematic of the three personalization architectures represented in the included studies. **(A)** Static neural-signature personalization: musical material is derived in advance from the participant’s own EEG and played back without further adaptation. **(B)** Real-time parametric closed loop: ongoing EEG continuously modulates parameters of pre-existing music, with the acoustic output feeding back to the listener. **(C)** Autonomically driven adaptation: heart rate variability (HRV) and electrodermal activity (EDA) guide continuous modulation. EEG, electroencephalography.

### 3.4 Musical material and listening conditions

Table 3 sets out the musical material used in each study, the acoustic parameters placed under algorithmic control, the state each intervention targeted, and the conditions under which listening took place.

Reporting of the musical stimulus itself is sparse. No study named a composer or a specific work. A genre or musical style is identifiable in five studies. These cover classical music (Takabatake et al., 2021), the Chinese five-tone framework and its Jue mode (Xu and Li, 2025), and culturally relevant commercial tracks matched to the individual listener (Hećimović et al., 2026). They also cover popular songs linked to the listener’s own past (Byrns et al., 2020; Sakakibara et al., 2025). Tempo was an explicitly controlled or reported parameter in three studies. It was aligned to footfall (Moens et al., 2014), driven by frontal alpha asymmetry (Ramirez et al., 2015), or used to select slow excerpts (Byrns et al., 2020). An intended emotional character was specified in five studies. It was given as a target emotional state (Miyamoto et al., 2022), as valence and arousal targets (Zhou et al., 2026), and as valence, arousal, and motivational tone (Wu et al., 2025). Byrns et al. (2020) chose excerpts for anxiety-reducing features, and Sakakibara et al. (2025) targeted nostalgia. In the remaining studies the musical material is described only by the procedure that produced it.

Listening conditions were similarly heterogeneous. Music was delivered around sleep in two studies, before bedtime in one (Gao et al., 2020) and overnight in the other (Hećimović et al., 2026). It was delivered during a clinical procedure in one study (Huang et al., 2016) and during an experimental anxiety induction in another (Yang et al., 2025). Three studies delivered music alongside a concurrent cognitive or driving task (Chiu and Ko, 2017; Wu et al., 2025; Zhu and Zhang, 2025), and one delivered it during walking and running, in the laboratory, on a treadmill, outdoors, and at an exhibition (Moens et al., 2014). Song and colleagues embedded delivery in everyday use over 12 weeks, and Byrns and colleagues delivered it in an immersive virtual-reality theatre. In the remaining eleven studies listening took place in a laboratory or clinic session, and no concurrent activity is described in the available record.

Where these attributes were reported, favorable outcomes accompanied classical material under alpha neurofeedback (Takabatake et al., 2021), Jue-mode five-tone material for anxiety (Xu and Li, 2025), and culturally relevant tracks for epileptiform activity (Hećimović et al., 2026). Tempo aligned to footfall accompanied gait synchronization (Moens et al., 2014), and tempo driven by frontal alpha asymmetry accompanied lower depression scores (Ramirez et al., 2015). Generation aimed at a specified emotional state was reported to induce that state (Miyamoto et al., 2022), and emotionally differentiated generated music was associated with different memory and workload effects in numerical and image-based tasks (Wu et al., 2025). Slow popular songs from the listener’s youth were reported alongside better attention and memory scores, without a control condition or statistical test (Byrns et al., 2020). Only one study compared one emotional character against another. Songs the system ranked most nostalgic for each listener raised ratings of nostalgia and well-being more than songs it ranked least nostalgic, and raised memory vividness in older listeners (Sakakibara et al., 2025). No study compared one genre or tempo against another, so none of the other outcomes can be credited to the attribute itself. By listening condition, favorable outcomes were reported during overnight sleep (Hećimović et al., 2026) and a clinical procedure (Huang et al., 2016). They were also reported during task performance and simulated driving (Chiu and Ko, 2017; Zhu and Zhang, 2025), during walking and running (Moens et al., 2014), and in virtual reality (Byrns et al., 2020). The pre-bedtime sleep result is contested (Gao et al., 2020), and no study compared the same music across conditions. Two consequences follow. Attributing an observed effect to a particular kind of music is not possible across most of this corpus, because the attribute carrying the attribution is usually unreported. Comparison across studies is also limited, since a twenty-minute seated session and an all-night playlist are different interventions delivered under different conditions.

### 3.5 Neurophysiological outcomes

#### 3.5.1 EEG oscillatory power

Findings converged on frontal and prefrontal effects but diverged by frequency band, consistent with the differing targets of each intervention. Yang et al. (2025) reported enhanced prefrontal theta oscillations accompanying attenuated anxiety induction, interpreting theta as the mechanism given established prefrontal theta involvement in anxiety and depressive state regulation. Takabatake et al. (2021) targeted alpha explicitly and confirmed significantly higher normalized alpha power under real relative to yoked random feedback. Ramirez et al. (2015) reported a decrease in relative left frontal alpha activity (*p* = 0.00008), interpreted as improvement in depressive state via the frontal alpha asymmetry framework. These are directionally consistent with the respective theoretical targets but are not directly comparable, since one study sought to increase alpha globally and another to decrease it lateralized. The Scopus-identified studies extend the frontal oscillatory pattern: customised brainwave music enhanced theta and alpha power and inter-regional functional connectivity relative to both cognitive behavioral therapy and no-intervention controls during orthodontic pain (Huang et al., 2016); five sessions of closed-loop personalized music neurofeedback increased frontal and temporal alpha power alongside microstate changes correlating with stress reduction (Hu et al., 2024); contingent narrow-band oscillator feedback normalized EEG relative to non-contingent listening (Fedotchev, 2018); and transformer-generated personalized melodies increased frontal alpha asymmetry fluctuation by 52.97% in a small feasibility sample (Wijethunge et al., 2024).

The Chinese-language studies extend the same pattern. After music neurofeedback delivered for 10 min daily over 14 days, the relative power of alpha, beta, and theta rhythms increased and delta relative power decreased. Sample entropy and complexity both rose significantly, while an unstimulated control group showed no significant change on any parameter (Li et al., 2020). Brain-wave music converted from gaming-state EEG was associated with reduced beta-band connectivity between prefrontal, frontal, parietal, bilateral temporal, and occipital regions, alongside the largest fatigue reduction among four conditions (Wang et al., 2023). Two studies found through the Wanfang search reported EEG-derived affect indices instead of band power. In Byrns et al. (2020), a frustration index was 0.69 before, 0.45 during and 0.51 after listening, and a valence index was 0.59, 0.54 and 0.75. In Sakakibara et al. (2025), EEG-decoded nostalgia was higher for songs ranked most nostalgic than for songs ranked least nostalgic in older adults (*p* = 0.003), and not in younger adults (*p* = 0.080).

The caution applied below to Gao et al. (2020) applies to these oscillatory findings as well. Ramirez et al. (2015) report *p* = 0.00008 for the change in left frontal alpha, and the estimate comes from six completers in an uncontrolled design with no correction for the number of electrodes and bands examined. The 52.97% increase in frontal alpha asymmetry fluctuation reported by Wijethunge et al. (2024) comes from ten participants with no control condition. Hu et al. (2024) report significant band-power and microstate change in five participants. Li et al. (2020) report significant change in the intervention group and none in an eight-person control group, and the two groups were not compared directly, so the contrast does not establish a difference between them. Byrns et al. (2020) report no statistical test and no control condition. The controlled findings carry the same limit. Yang et al. (2025), Huang et al. (2016), Takabatake et al. (2021) and Wang et al. (2023) each come from a single study by one group, so their oscillatory and connectivity results are correlates awaiting replication and not demonstrated mechanisms. Sakakibara et al. (2025) is also a single study, and its decoder both measured nostalgia and steered song selection. Part of the difference between its conditions is therefore built into the design. In each case the direction of the finding is consistent with the study’s stated target, and the precision implied by the reported statistics exceeds what the designs support.

#### 3.5.2 Sleep architecture and delta power

Gao et al. (2020) reported that delta power spectral density decreased in the slow-wave-sleep brain-wave music group while increasing in both the REM-derived music and white noise groups [*F*(2,31) = 7.909, *p* = 0.005], with sleep efficiency ameliorated in the SWS group [*t*(10) = −1.943, *p* = 0.076] and reduced frontoparietal connectivity negatively correlated with sleep latency (*r* = −0.527, *p* = 0.064). This finding requires careful handling, because its interpretation has been formally contested. Johnson and Durrant (2021), in a published commentary, argued that reduced delta power during slow-wave sleep is more plausibly indicative of impairment than benefit, noting that the reduction occurred alongside unchanged slow-wave sleep duration and that the authors’ own concurrent findings of lower sleep latency and higher sleep efficiency are associated with increased rather than decreased homeostatic sleep pressure. We report the original finding and the dispute together, and do not treat the delta result as supporting benefit. Notably, several of the supporting statistics in the original report fall short of conventional significance thresholds (*p* = 0.076, *p* = 0.073, *p* = 0.064).

#### 3.5.3 Epileptiform activity and clinical neurophysiology

The clearest clinical neurophysiological signal in the corpus comes from Hećimović et al. (2026), in which all-night personalized playlists, selected by the X-System computational model to match audified healthy-hemisphere EEG, reduced epileptiform spikes by a mean of 28.42% in 19 children with epilepsy (28.46% focal, 28.33% generalized), with accompanying improvements in REM sleep and quality of life. The design is pre–post without a randomized control, so regression to the mean and expectancy cannot be excluded, but overnight video-EEG spike counting is not plausibly participant-reportable, making this among the more expectancy-resistant endpoints in the corpus. The finding is provisional on that basis. A pre–post nonrandomized trial in nineteen children establishes feasibility and a plausible signal, and it cannot establish efficacy.

#### 3.5.4 Event-related potentials

Zhou et al. (2026) reported significant P200 activation differences in left parietal and occipital regions and N400 differences in prefrontal regions across music stimulus types relative to white noise (both *p* < 0.001) in 68 older adults. These findings indicate that algorithmically selected music is associated with cortical responses distinguishable from noise, but the comparator does not permit attribution to the personalization procedure.

#### 3.5.5 Autonomic outcomes

Only one included study reported autonomic endpoints. Xu and Li (2025) found significantly increased high-frequency HRV and significantly decreased skin conductance level following a single 20-min session of HRV- and EDA-guided adaptive music (both *p* < 0.001), concordant with a large reduction in interviewer-administered HAMA scores. The concordance of subjective and autonomic measures is notable, but the single-group design cannot separate the effect of the adaptive algorithm from that of music exposure, rest, or expectancy. The authors state this limitation explicitly. At the opposite end of the scale spectrum, a 12-week controlled deployment of a data-driven AI music framework in 500 university students reported 25% greater stress reduction than a non-personalized control, supported by heart rate variability biomarkers (Song et al., 2025), and HRV-guided machine-learning music selection improved task performance without increasing mental workload in a wearable-sensing paradigm (Chiu and Ko, 2017).

### 3.6 Behavioral and cognitive outcomes

Objective behavioral and cognitive outcomes were more broadly represented in the expanded corpus. Takabatake et al. (2021) found greater short-term memory improvement among participants whose alpha power increased in response to real feedback. Hu et al. (2024) reported significantly improved response inhibition on the Stroop task after five neurofeedback sessions. Context-aware AI-generated music improved spatial memory retention and navigation efficiency relative to fixed music and silence in simulated driving (Zhu and Zhang, 2025), and emotionally differentiated AI-generated background music modulated memory performance in a task-dependent manner (Wu et al., 2025). In the motor domain, beat-to-footfall phase alignment produced strong increases in spontaneous gait synchronization relative to a random playlist (Moens et al., 2014). Byrns et al. (2020) selected music from each participant’s EEG response and delivered it in one virtual-reality session. Older adults with subjective cognitive decline then improved on attention (1.91–6.59%) and memory (6.14–36.84%) exercises, with no control condition or statistical test. No study reported follow-up assessment beyond the immediate or short post-intervention period; Ramirez et al. (2015) explicitly note the absence of follow-up as a limitation. In seven studies, affective self-report measures were reported alongside objective measures: state anxiety (Yang et al., 2025), HAMA (Xu and Li, 2025), BDI (Ramirez et al., 2015), DASS-21 (Hu et al., 2024), the Self-Rating Depression Scale (Li et al., 2020), ratings of nostalgia and well-being (Sakakibara et al., 2025), and pain VAS (Huang et al., 2016). In every case but one the two moved concordantly, which is reassuring but does not by itself distinguish a genuine intervention effect from a shared response to expectancy. The exception is the younger group in Sakakibara et al. (2025), where self-reported nostalgia rose and EEG-decoded nostalgia did not differ significantly (*p* = 0.080).

### 3.7 Comparative effects of personalization

Four causal questions are separated here, because the literature tends to run them together. First, does music exposure produce an effect at all? Second, does personalization to the individual add benefit beyond fixed music? Third, does real-time contingent adaptation add benefit beyond non-contingent delivery of the same material? Fourth, does algorithmically generated music add benefit beyond existing music? A study can only address the question its comparator is built for. Of the 21 included studies, eight carry a comparator capable of isolating an algorithmic component from music exposure, and those eight are distributed unevenly across the four questions.

The first question is already well answered. Music exposure produces effects on affect, cognition, and autonomic state, and a large meta-analytic literature already supports that conclusion (Zhao et al., 2024; Lu et al., 2025; Tang et al., 2025). Studies in this corpus comparing algorithmic music against silence, white noise, or no intervention speak to that question and to no other, whatever their authors conclude.

The second question, whether personalization to the individual adds benefit beyond fixed music, is addressed adequately by one study. Yang et al. (2025) allocated participants to brain-wave music generated from their own resting-state EEG, to self-selected preferred music, or to silence. Anxiety induction was attenuated in the brain-wave music arm alone, and the preferred-music arm showed no significant change. Preferred music is by construction well matched to the individual, so the contrast is informative about content personalization specifically. Because the brain-wave music was produced by sonification and the comparator was existing music, the same contrast also bears on the fourth question, and the design cannot separate the two. Song et al. (2025) also compared a personalized against a non-personalized condition, and the intervention was multicomponent, so personalization cannot be separated there from engagement and dosage. Sakakibara et al. (2025) bears on the question less directly. The same system played songs it ranked most nostalgic for the listener in one condition and songs it ranked least nostalgic in the other. Ratings of nostalgia and well-being were higher in the nostalgic condition in both age groups. The comparator was chosen to be the least suitable music and the outcomes were mainly self-reported. The contrast therefore shows that the targeting worked, and not that it adds benefit beyond well-chosen fixed music.

The third question, whether real-time contingency adds benefit beyond non-contingent delivery, is addressed by four studies. Takabatake et al. (2021) held the music, the noise superimposition, and participant expectation constant, and removed only the neural contingency by yoking the feedback. Normalized alpha power was significantly higher under real feedback. Fedotchev (2018) reached the same architecture-level conclusion within participants, by comparing contingent oscillator-driven music against non-contingent listening. Miyamoto et al. (2022) compared generation conditioned on the participant’s EEG-predicted emotional state against the identical generator running unconditioned, and conditioned generation induced target emotions more effectively. Moens et al. (2014) compared phase-and-tempo-locked alignment against a random playlist, isolating contingency for gait synchronization.

The fourth question, whether algorithmically generated music adds benefit beyond existing music, is addressed directly by two studies. Generated music is taken here to include rule-based sonification as well as machine-learning composition, since both produce new material instead of drawing on existing recordings. Wang et al. (2023) randomized 75 participants to gaming-state brain-wave music, eye-open resting-state brain-wave music, non-brain-wave music, or silence after an experimental fatigue induction. All three music conditions reduced fatigue, and the gaming-state brain-wave music was followed by the largest fatigue reduction. This pattern is consistent with a possible benefit of the EEG-derived material over ordinary music. Zhu and Zhang (2025) compared context-adaptive AI-generated music against fixed music and silence, with the adaptive condition outperforming both on spatial memory and navigation. Because the AI-generated music in that study was also adapted in real time, the comparison cannot separate generation from contingency.

All eight comparator studies reported results in the direction of the algorithmic condition. The convergence is worth noting and it does not establish a mechanism. The eight test different architectures, use different comparators, and measure different outcomes in different populations, and they address three different causal questions between them. One of the eight bears on content personalization and generated material together, one on content personalization alone, four on real-time contingency, and two on generated material. No specific finding among them has been independently replicated, and the largest single experiment enrolled 119 participants (Moens et al., 2014). The results are best characterized as preliminary evidence consistent with a graded contribution of personalization and adaptation, and as hypothesis-generating for the trials set out under implications for research and practice.

The remaining thirteen studies cannot support inference about the algorithmic component. Gao et al. (2020), Zhou et al. (2026), and Wu et al. (2025) compared against white noise or between stimulus conditions. Huang et al. (2016) compared against cognitive behavioral therapy and no intervention, which informs clinical positioning and is silent on whether the algorithmic derivation mattered. Li et al. (2020) compared against an unstimulated control, so the comparison speaks to music exposure and not to the neurofeedback contingency. Song et al. (2025) compared against a non-personalized control within a multicomponent intervention. The remainder were uncontrolled or within-subject pre–post designs (Ramirez et al., 2015; Chiu and Ko, 2017; Byrns et al., 2020; Hu et al., 2024; Wijethunge et al., 2024; Xu and Li, 2025; Hećimović et al., 2026). The pre-specified subgroup contrast of neural against autonomic personalization remains underpowered, with only two autonomically driven studies. Meta-analysis was not possible for any outcome, because no two studies reported a common outcome measure under comparable comparators.

### 3.8 Risk of bias

Risk of bias was rated high or serious for all included studies. No study blinded participants, and the difficulty of blinding is intrinsic to this intervention. More consequentially, only Takabatake et al. (2021) reported single-blind conditions, no study reported blinded analysts, and no study measured participant expectancy or beliefs about AI despite the technological framing of the intervention. Six studies had no control condition. Attrition was a concern in Ramirez et al. (2015), where four of ten enrolled participants withdrew and analysis proceeded on the six completers without intention-to-treat handling. Reported sample sizes were predominantly small, with a median of 22 and a range of 5 to 500, the upper bound contributed by a single multicomponent deployment. Two engineering-venue reports did not state a sample size in the available record, and no study reported an *a priori* power calculation. Reporting of the personalization algorithm was insufficient for independent replication in roughly half of the studies. The sonification mappings of Gao et al. (2020) and Yang et al. (2025) are described at the level of the power-law principle and not as reproducible parameters, Xu and Li (2025) do not specify the modulation rules connecting the autonomic signal to musical output, the spectral classification rule of Li et al. (2020) is not given in reproducible form, and several engineering-venue reports specify architectures without parameters. Gao et al. (2020) additionally illustrates a selective-interpretation risk, since several supporting statistics fell short of conventional thresholds yet were incorporated into a positive conclusion subsequently disputed in the published literature.

## 4 Discussion

### 4.1 Summary of principal findings

Three findings emerge from this review. First, algorithmically personalized music interventions are consistently associated with measurable neurophysiological change. Frontal and prefrontal oscillatory changes were reported consistently across studies using EEG, and autonomic and event-related potential effects were reported where measured. Second, the eight studies employing an adequate comparator all favored the algorithmic condition. The convergence is preliminary evidence that the effects exceed those of the comparator conditions. Yang et al. (2025) found benefit for brain-wave music and not for self-selected preferred music, Takabatake et al. (2021) isolated the closed-loop contingency by yoking feedback, and Wang et al. (2023) found the largest fatigue reduction for EEG-derived music in a comparison that included ordinary music. These designs are small, they address different causal questions, and none has been independently replicated. Third, the evidence base is methodologically immature to a degree that precludes clinical inference. Roughly two-thirds of the included studies cannot distinguish the algorithmic component from music exposure, and none measured expectancy in an intervention whose framing invites it. Reported samples are predominantly small, two reports do not state a sample size, and one prominent finding has been publicly contested on interpretive grounds. GRADE certainty was very low for every outcome domain in Table 4.

A distinction should be held throughout. Neurophysiological change, behavioral change, and clinical benefit are separate claims with separate evidential requirements. This corpus reports neurophysiological change most often, behavioral change less often, and clinical benefit least often, and a change in EEG oscillatory power does not by itself demonstrate that a person is better.

A structural observation cuts across these findings. The design space defined in Table 2 is unevenly populated, and its most theoretically promising cell, generative individualized content under real-time neural sensing, is occupied by a single small emotion-induction validation (Miyamoto et al., 2022). Studies that personalize content to the individual’s neural signature otherwise do not adapt in real time; studies that adapt in real time otherwise modulate parameters of pre-existing material or select from a corpus. The intervention that the theoretical accounts most strongly motivate has been demonstrated as feasible but has not been tested in humans against any clinical or health outcome, and no specific finding anywhere in the corpus has been independently replicated.

### 4.2 Mechanistic interpretation

The mechanistic question raised by this literature is whether algorithmic personalization engages neural processes that fixed music does not, or whether it simply delivers a better-matched instance of the same process. These alternatives make different predictions. If personalization operates by improving stimulus–state congruence, its effects should be continuous with those of well-chosen preferred music and largest where individual variability in musical response is greatest. If instead closed-loop systems engage a distinct mechanism, namely recursive coupling between neural state and acoustic output analogous to that proposed for neurofeedback learning (Sitaram et al., 2017), then real-time systems should outperform preference-matched controls, and the advantage should scale with reduced adaptation latency rather than with the quality of the initial match.

Four of the comparator studies bear on this question, and they point in the same direction while testing different architectures. Yang et al. (2025) bears on the matching side of the question. Music derived from the participant’s own neural signature outperformed self-selected preferred music, and a pure congruence account does not readily explain the contrast, since preferred music is by construction well matched to the individual. Because that music was static, the result concerns what the music contains and not the closed loop. Three studies isolate contingency on a neural signal. Takabatake et al. (2021) found that identical music with identical noise superimposition produced higher alpha power when the noise was contingent on neural activity than when it was not. Fedotchev (2018) reports a contingency effect in the same direction with a different signal definition and design, and Miyamoto et al. (2022) shows the same pattern for generative systems, since conditioning on the listener’s neural state improved emotion induction over the identical generator running unconditioned. These three results are more consistent with the mechanistic-distinctness account than with the better-matching account. None is decisive. The combined evidence remains small and unreplicated, and the mechanisms set out below should be read as plausible hypotheses that the current designs cannot adjudicate.

Existing theoretical accounts do not fully adjudicate. Predictive-processing frameworks hold that music perception, emotion, and reward rest on the brain’s fundamental predictive machinery (Friston, 2010; Koelsch et al., 2019; Vuust et al., 2022), with musical pleasure arising from the interplay of expectation and its violation, implying that endlessly non-repeating generative material may sustain engagement fixed recordings cannot, but also that fully state-congruent music risks eliminating the productive prediction error on which musical reward depends (Sayal et al., 2025). Entrainment accounts predict that stimuli locked to endogenous oscillatory activity should modulate that activity more effectively than stimuli that are not, favoring neural-signal-driven architectures specifically (Ingendoh et al., 2023; Ding et al., 2025). Converging support for the closed-loop mechanism comes from the broader alpha-neurofeedback literature, where upper-alpha training improves cognitive performance (Zoefel et al., 2011; Escolano et al., 2011) and auditory alpha feedback embedded in relaxing music raises alpha power across sessions (Phneah and Nisar, 2017); conversely, theta-range auditory beat stimulation added to calm music produced no incremental anxiolytic benefit over music alone in a large randomized trial (Mallik and Russo, 2022), a caution that not every physiologically motivated acoustic manipulation earns its complexity. The empty design-space cell identified in Section 3.3 is precisely where these accounts make divergent predictions, which is a further reason to prioritize it experimentally.

### 4.3 Methodological limitations of the evidence base

The most consequential limitation concerns control conditions. Where a study compares AI-generated music against silence, white noise, no intervention, or treatment as usual, the resulting effect estimate speaks to the value of music, not to the value of personalization; the design cannot distinguish the two. Roughly two-thirds of the included studies fall into this category, and some engineering-venue studies compound the problem with incomplete reporting of comparators or sample sizes. Attribution to the algorithm requires an active comparator matched for musical exposure, and ideally for novelty as well, since generative systems produce unfamiliar material by construction and unfamiliarity is itself an active variable. The yoked-feedback design of Takabatake et al. (2021) is the strongest available solution and should be adopted more widely; consensus recommendations for music-based rehabilitation trials make the same demand for exposure-matched active comparators (Grau-Sánchez et al., 2022).

Expectancy presents a related and equally structural problem. Participants who are told that a system is generating music in real time from their own brain activity have strong grounds to expect benefit. The technological framing of these interventions may therefore amplify placebo response relative to conventional music therapy. Several distinct routes are at work and the corpus separates none of them. Novelty is one, since generative systems produce unfamiliar material by construction, and unfamiliarity alters attention and arousal on its own. Beliefs about artificial intelligence are another, and they vary widely in the general population, so two participants given the same intervention may hold very different priors about whether it works. Engagement is a third, since a system that visibly responds to the listener invites sustained attention in a way a fixed recording does not. Authors of the feasibility work in this area acknowledge that single-group pre–post designs cannot separate intervention effects from expectancy, Hawthorne effects, and spontaneous fluctuation (Xu and Li, 2025). Objective neurophysiological endpoints partially mitigate the concern and do not resolve it, since expectancy has measurable autonomic and cortical correlates of its own. No study in the corpus measured expectancy, and none reported the wording used to describe the intervention to participants.

Technical heterogeneity is extreme and under-reported. Systems differ in sensing hardware, electrode count, feature extraction, mapping function, and generative approach. As a result, two studies described identically as EEG-based adaptive music may share almost no implementation detail. The corpus includes 4-electrode consumer headbands and full research-grade montages, power-law sonification and simple parametric mapping. Where the mapping function from physiological state to musical parameter is not fully specified, replication is impossible. Finally, the Gao et al. (2020) case illustrates a broader interpretive risk: in a young field with strong priors that the intervention works, neurophysiological findings admit of multiple readings, and the published commentary disputing the delta-power interpretation (Johnson and Durrant, 2021) should be read as a caution about direction-of-benefit assumptions rather than as a criticism of that study alone.

### 4.4 Listener characteristics as moderators

Listener characteristics are the other variable this corpus leaves unmeasured. Musical response is not uniform across listeners, and the moderators are well documented outside this literature. Age changes the response to the same material. Younger listeners showed larger self-reported arousal change and found meditative binaural music more calming than low-arousal classical music, and older listeners rated the low-arousal classical music as the most comforting (Lee-Harris et al., 2018). Men scored higher than women on cognitive regulation through music listening (Verma, 2021). In a crossmodal priming study, listening to music raised women’s but not men’s ratings of facial attractiveness, and high-arousal music produced the largest effect (Marin et al., 2017). One EEG study examined how responses to identical algorithmically generated material varied with listeners’ sex and musical training (Huang et al., 2023). It is not part of the corpus because music served there as a stimulus for comparing EEG feature-extraction methods, not as an intervention. Neurohumoral state is a further candidate moderator. Menstrual cycle phase has been examined as a factor in whether music acts as an anxiolytic or a stressor for female professional musicians (Bazanova, 2003), and it alters mood states (Ghazel et al., 2022). The benefit of music for short-term exercise performance (Ghazel et al., 2022) and its effect on attractiveness judgements (Marin et al., 2017) did not differ across cycle phases. Neither study tested whether cycle phase alters the anxiolytic effect of music, which many personalized systems target, and no included study examined cycle phase at all. Cultural background shapes which material is experienced as calming, and one study in this corpus treated cultural relevance as a selection constraint (Hećimović et al., 2026).

None of the included studies reported a validated measure of musical sophistication, and only one analyzed outcomes by age group, finding larger gains in nostalgia, well-being and memory vividness in older than in younger listeners (Sakakibara et al., 2025). None analyzed outcomes by sex or musical background. Fifteen of the twenty-one studies were conducted in East Asian populations, and generalization beyond those settings is untested. The omission matters more here than in conventional music therapy. A system that tailors music to the individual is making a claim about individual differences, and a literature that does not measure the best-documented individual differences cannot evaluate that claim. Measuring them would also make the comparator problem more tractable, since a moderator predicting who responds would help separate a specific mechanism from a general expectancy effect.

### 4.5 Limitations of this review

Several limitations qualify this review. The Chinese-language search was conducted in one pass without independent duplicate screening, so Chinese-language coverage is less complete than the English-language coverage. Four of the twenty-one included studies came from the Chinese-language searches. Eleven of the twenty-one studies originate from Chinese institutions, so further Chinese-language work almost certainly exists beyond what these searches retrieved. Sample size and effect data were not reported in the available records of two included conference or engineering-venue reports, and the corresponding cells of Table 5 are marked as not reported. Conclusions rest principally on the nineteen studies with complete reporting. One of the eight comparator studies, Zhu and Zhang (2025), is among the two without a reported sample size. Grey literature and non-peer-reviewed preprints were excluded, which in a fast-moving technical field may exclude current work. Theses and dissertations were excluded by the *a priori* criteria, and the Chinese-language searches showed that a substantial body of relevant work sits in that category. Terminological inconsistency across clinical and engineering literatures may have caused eligible studies to be missed despite the breadth of the search strategy. The concepts of interest are variously described as adaptive music, generative music therapy, musical neurofeedback, brain-wave music, sonification, and affective music generation, and no controlled vocabulary spans them. Screening and extraction were performed by one reviewer throughout, without independent duplicate assessment, raising the risk of selection bias and of inconsistent application of the eligibility criteria. Meta-analysis was not possible for any outcome, so all quantitative conclusions rest on narrative synthesis of individual studies. A registered systematic review with dual independent screening will become worthwhile as the primary literature grows.

**Table 5:** Characteristics of included studies.

Study	Country/design	Sample	Intervention & architecture	Comparator	Objective outcomes	Key findings
Yang et al. (2025)	China; two-phase, randomized allocation to three groups	*n* = 70 (BWM 30, PMC 20, SC 20); healthy adults	Brain-wave music generated from participant’s own resting-state EEG; neural sensing, static personalization, rule-based sonification	Self-selected preferred music (PMC); silent control (SC)	EEG spectral power and connectivity; State Anxiety Inventory	Anxiety increase during second induction significantly attenuated in BWM only (*t* = 2.377, *p* < 0.05); no significant change in PMC or SC. Enhanced prefrontal theta oscillations and prefrontal–multisensory connectivity identified as neural correlate.
Gao et al. (2020)	China; randomized three-group, 6-day exposure	*n* = 33 (SWS 11, REM 13, WN 9); young adults	Brain-wave music derived from sleep-stage EEG (amplitude, period, average power mapped to pitch, duration, volume, timbre via power law); 20 min pre-bedtime	REM-derived brain-wave music; white noise	Polysomnography; EEG power spectral density; functional connectivity	Delta PSD decreased in SWS group but increased in REM and WN groups [*F*(2,31) = 7.909, *p* = 0.005]. Sleep efficiency ameliorated in SWS group [*t*(10) = −1.943, *p* = 0.076]. F3–C3 connectivity decreased [*t*(10) = 1.969, *p* = 0.073], negatively correlated with sleep latency (*r* = −0.527, *p* = 0.064). Interpretation contested—see Section 3.5.2
Takabatake et al. (2021)	Japan; single-blind randomized crossover, ≥4 weeks per arm	*n* = 10 healthy adults	Music-based auditory alpha neurofeedback: white noise superimposed on classical music, noise level inversely correlated with normalized alpha power; 4-electrode wearable EEG; neural sensing, real-time adaptation, parametric modulation	Yoked random feedback (RF) within the same participants	Normalized EEG alpha power; cognitive battery incl. Short-term memory	Normalized alpha power significantly higher under real feedback than random feedback. Greater short-term memory improvement in participants whose alpha power increased in response to real feedback.
Xu and Li (2025)	China; single-group pre–post feasibility	*n* = 40 university students with moderate anxiety (M age 21.5)	AI-driven five-tone (Jue tone) modulation with real-time adaptation guided by HRV and EDA; autonomic sensing, real-time adaptation, parametric modulation; single 20-min session	None (within-subject pre–post)	HF-HRV; skin conductance level; HAMA (interviewer-administered)	HAMA reduced from 18.2 (SD 2.5) to 11.5 (SD 2.1); mixed-effects model *t*(78) = −21.84, *p* < 0.001. HF-HRV significantly increased and SCL significantly decreased (both *p* < 0.001). Authors note design cannot exclude expectancy or Hawthorne effects.
Ramirez et al. (2015)	Spain; uncontrolled clinical pilot, 10 sessions over 5 weeks	*n* = 10 enrolled (9F, mean age 84); 6 completed pre/post assessment	Musical neurofeedback: participant-preselected pieces with loudness driven by frontal beta/alpha ratio (arousal) and tempo by frontal alpha asymmetry (valence); neural sensing, real-time adaptation, parametric modulation	None (within-subject pre–post)	EEG relative alpha activity; BDI	Mean BDI improvement 17.2% (SD 1.3) in six completers. Significant decrease in relative alpha activity in left frontal lobe (*p* = 0.00008). Four participants withdrew for health reasons; no follow-up conducted.
Zhou et al. (2026)	China; cross-sectional experimental	*n* = 68 older adults aged 60–75	Multimodal Fusion-Adaptive Wellness Interaction model mapping music parameters to valence-arousal states; multimodal sensing, session-level adaptation, algorithmic selection	White noise condition	EEG event-related potentials (P200, N400, P300); frequency-band analysis	All four music stimulus types differed significantly from white noise in EEG activation. P200 activation in left parietal and occipital regions (P7, P3, O1, O2; *p* < 0.001); N400 differences in prefrontal region (*p* < 0.001).
Huang et al. (2016)	China; three-arm RCT, 1 week	*n* = 36 orthodontic patients (12/arm)	Customised brainwave music composed from each participant’s baseline resting EEG; neural sensing, static personalization, rule-based sonification	Cognitive behavioral therapy; no-intervention control	EEG (connectivity, complexity, band power); VAS pain	BWM and CBT groups reported significantly lower pain than control on days 1–4. BWM additionally showed improved inter-regional functional connectivity, lower EEG complexity, and enhanced theta and alpha power vs. CBT and control, clustered in prefrontal, frontal, parietal, occipital regions.
Fedotchev (2018)	Russia; within-subject controlled pilot, 2 sessions	*n* = 16 workers with stress complaints	Musical neurofeedback driven by individually identified narrow-band EEG oscillators detected in real time; neural sensing, real-time adaptation	Music listening without feedback contingency (within-subject)	EEG; stress symptom measures	EEG normalization and reduction of stress indices reported under contingent feedback relative to non-contingent listening.
Miyamoto et al. (2022)	Japan; within-subject controlled experiments	*n* = 20 (10 M/10F)	Generative music system conditioned on emotions predicted from EEG in real time, with iso-principle trajectory from current toward target emotional state; neural sensing, real-time adaptation, ML-generative content	Music generation not conditioned on participant emotion	EEG-predicted emotional state; prediction RMSE	Iso-principle and emotion-conditioned generation induced target emotions more effectively than generation not based on participants’ emotions (*p* < 0.016); meta-learning reduced EEG training-data requirements.
Hu et al. (2024)	China; single-group feasibility, 5 sessions	*n* = 5 university students	Closed-loop neurofeedback with personalized music selection; neural sensing, real-time adaptation	None (within-subject pre–post)	EEG band power and microstates; Stroop task; DASS-21	Stress-related scores decreased and response inhibition improved significantly; frontal and temporal alpha power increased; microstate C coverage/duration decreased and microstate D duration increased, correlating with stress reduction.
Wijethunge et al. (2024)	Sri Lanka; single-group feasibility	*n* = 10 workers	Transformer-based generative model producing personalized music from EEG-identified preferred song segments; neural sensing, static personalization, ML-generative content	None (within-subject)	Frontal alpha asymmetry (FAA)	52.97% increase in FAA fluctuation with generated melodies; mean satisfaction 3.9/5. Preliminary; intervention trials planned.
Hećimović et al. (2026)	Croatia/UK; pre–post clinical study, 3 weeks	*n* = 19 children with epilepsy (5–17 y)	X-System computational model selecting culturally relevant tracks matched to audified contralateral healthy-hemisphere EEG; all-night personalized playlist; neural-derived, static algorithmic selection	Within-subject baseline (video-EEG + PSG before treatment)	Overnight video-EEG epileptiform spikes; polysomnography; QOLIE-31/ELDQOL	Mean epileptiform spike reduction 28.42% (focal 28.46%, generalized 28.33%); REM sleep and quality-of-life improvements reported.
Chiu and Ko (2017)	Taiwan; within-subject experiments	n/r	Machine-learning music selection guided by wearable HRV sensing and learned preference; autonomic sensing, real-time algorithmic selection	Task performance without HRV-guided selection	Task/learning performance; mental workload	HRV-guided selection improved task performance and satisfaction without increasing mental workload.
Moens et al. (2014)	Belgium; 4 experiments (lab, treadmill, open air, exhibition)	*n* = 10–119 per experiment	D-Jogger adaptive player sensing walking/running tempo and phase, aligning musical beat to footfall in real time; behavioral sensing, real-time parametric modulation	Random playlist; between-strategy comparisons	Step-beat phase synchronization	Phase-and-tempo-locked strategies produced a strong increase in synchronized steps versus random playlist; tempo-only matching produced minor increases.
Zhu and Zhang (2025)	China; within-subject simulated driving	n/r	Context-aware AI-generated music adapted in real time to road conditions; contextual sensing, real-time ML-generative content	Normal (fixed) music; silence	Spatial memory task; route accuracy; reaction time; error rate	Context-aware AI music significantly improved spatial memory retention and navigation efficiency versus fixed music and silence.
Wu et al. (2025)	China; within-subject experiments	*n* = 17 learners	AI-generated background music with parametrically controlled emotional attributes (valence, arousal, motivational tone); condition-level generation, no individual sensing	Between-condition contrasts of AI-music emotion profiles	Numerical and visual memory performance; workload; physiological state	Task-dependent effects: emotionally differentiated AI music modulated memory performance and workload differently across numerical and image-based paradigms.
Song et al. (2025)	China; controlled trial, 12 weeks	*n* = 500 university students	Data-driven AI framework delivering individualized, dynamically adjusted music interventions from interaction logs and physiological signals; multimodal sensing, session-level adaptation	Non-personalized control group	Heart rate variability (digital biomarker); psychological scales	25% greater stress reduction and 16% greater increase in positive affect vs. control, supported by HRV digital biomarkers.
Li et al. (2020)	China; controlled pre–post, 14 days	*n* = 24 (16 intervention, 8 control); university students with sub-threshold depression	Music neurofeedback using decompression music selected by spectral classification; neural sensing, real-time adaptation, algorithmic selection	Unstimulated control group	EEG relative power, sample entropy, complexity; Self-Rating Depression Scale (SDS)	Relative power of alpha, beta and theta rhythms increased and delta decreased in the intervention group. Sample entropy and complexity increased significantly (*p* < 0.05). No feature parameter changed significantly in the control group. SDS scores decreased after stimulation.
Wang et al. (2023)	China; randomized four-group comparison	*n* = 75 healthy university students	Brain-wave music converted from EEG recorded in a gaming state and in an eye-open resting state; neural-derived, condition-level generation, rule-based sonification, static playback	Non-brain-wave music; silence	EEG power and brain-network connectivity; fatigue self-rating scale	All three music conditions reduced fatigue after an experimental fatigue induction, and gaming-state brain-wave music produced the largest effect. Gaming-state brain-wave music reduced beta-band connectivity between prefrontal, frontal, parietal, bilateral temporal and occipital regions.
Byrns et al. (2020)	Canada; single-group pre–post, single session	*n* = 19 older adults with subjective cognitive decline (mean age 72.3)	Song selected by an EEG agent from the participant’s emotional response to eight 30-s excerpts, then replayed in a virtual-reality theatre; neural sensing, static personalization, algorithmic selection	None (within-subject pre–post)	EEG-derived frustration and valence indices; attention and memory exercises; Positive and Negative Affect Schedule	Frustration index 0.69 before, 0.45 during and 0.51 after listening; valence index 0.59, 0.54 and 0.75. Mean improvement of 1.91–6.59% on attention and 6.14–36.84% on memory exercises. No statistical tests reported.
Sakakibara et al. (2025)	Japan; within-subject comparison of two feedback conditions	*n* = 33 (17 older adults aged 70–87, 16 younger adults aged 18–47)	Nostalgia Brain-Music Interface: an in-ear EEG decoder updates a song-recommendation model every 20 s; neural sensing, sub-minute closed-loop adaptation, algorithmic selection	Songs the same system ranked least nostalgic (non-nostalgic condition)	EEG-decoded nostalgia; ratings of nostalgia, well-being and memory vividness	Ratings of nostalgia, well-being and memory vividness higher in the nostalgic condition in older adults (all *p* < 0.001). In younger adults nostalgia (*p* = 0.003) and well-being (*p* = 0.002) higher, memory vividness not significant (*p* = 0.057). Decoded nostalgia higher in older adults (*p* = 0.003), not in younger adults (*p* = 0.080).

n/r, not reported. BWM, brain-wave music; PMC, preferred music control; SC, silent control; SWS, slow-wave sleep; REM, rapid eye movement; WN, white noise; RF, random feedback; HRV, heart rate variability; EDA, electrodermal activity; HF-HRV, high-frequency heart rate variability; SCL, skin conductance level; HAMA, Hamilton Anxiety Rating Scale; BDI, Beck Depression Inventory; PSD, power spectral density.

### 4.6 Implications for research and practice

The design requirements for the next generation of studies follow directly from the limitations above. We propose that trials of algorithmically personalized music interventions should, at minimum:

1 Employ an active comparator matched for musical exposure, duration, and novelty, including preference-matched fixed music where personalization is the question under test. Yoked or randomized feedback generated by the same system is the most informative control, since it holds the generative process and participant expectation constant while removing only the adaptive contingency.
2 Report the personalization architecture in sufficient detail for replication: sensing hardware and electrode montage, sampling and preprocessing parameters, feature extraction, the mapping function from physiological state to musical parameter, adaptation latency, and the generative model with the size and sources of its training data.
3 Include at least one objective neurophysiological or performance endpoint alongside self-report, and report both, including where they diverge.
4 Measure participant expectancy and prior beliefs about artificial intelligence, and report the technological framing used in consent and instruction procedures verbatim.
5 Characterize participants’ musical background using a validated instrument, since musical sophistication moderates neural response to music and is likely to interact with personalization; the Gold-MSI is a validated and freely available option (Müllensiefen et al., 2014). No included study did so.
6 Pre-register hypotheses, primary outcomes, and analysis plans, with particular attention to the frequency bands and connectivity metrics to be analyzed, given the analytic flexibility inherent in EEG.
7 Replicate independently and report outcomes on a common footing. No finding in this corpus has been reproduced by a group other than the one that produced it, and no two studies reported the same outcome measure under comparable comparators. Agreement on a small core set of neurophysiological, behavioral, and clinical endpoints would make quantitative synthesis possible.
8 Report the musical material and the listening conditions. Genre or style, tempo, intended emotional character, the acoustic parameters under algorithmic control, the listening posture or concurrent activity, and the session dose should all be stated. Outcomes should also be analyzed by age, sex, and musical background where the sample permits.

Beyond these, an instructive model exists in the motor domain, where a proof-of-concept digital therapeutic automated the progressive individualization of rhythm-based gait training after stroke (Hutchinson et al., 2020). That work demonstrates that algorithmic individualization can be specified, automated, and reported reproducibly. For the affective and cognitive domains reviewed here, the near-empty cell in the design space (Figure 1) is the clearest research priority: a clinically anchored trial combining generative individualized content with real-time closed-loop adaptation, extending the feasibility architecture of Miyamoto et al. (2022) to health outcomes, compared against both non-adaptive output from the same generative system and preference-matched fixed music, would test the field’s central hypothesis directly. Longer follow-up is also required; no included study assessed durability beyond the immediate post-intervention period.

One design would be particularly informative, and it follows from the comparator problem set out above. Participants would be randomized between music personalized by a human expert and music generated or selected algorithmically for the same individual, with a third arm receiving preference-matched fixed music. Exposure duration, familiarity, novelty, and the emotional characteristics of the material would be held constant across arms. The framing given to participants would be recorded verbatim and measured as expectancy. Such a design separates the contribution of the algorithm from the contribution of personalization as such, and it tests whether an algorithm adds anything to what a skilled human already does.

For clinical practice, the evidence does not support deployment. Three claims should be kept apart. Technology feasibility asks whether a system can be built and run with participants, and this corpus establishes feasibility for every architecture it describes. Proof of principle asks whether the system moves a measurable variable in the intended direction under controlled conditions, and the corpus supports that for several architectures, most securely for oscillatory outcomes under closed-loop delivery. Clinical efficacy asks whether patients are better off, and the corpus does not support that for any indication. The studies are proof-of-concept and feasibility work with a median sample of twenty-two participants, no blinded outcome assessment, no expectancy control, and no replication of any specific finding. The epilepsy result is the closest the corpus comes to a clinical endpoint, and it rests on a single pre–post nonrandomized trial in nineteen children. What the evidence does support is continued and better-designed investigation. Claims of clinical readiness in commercial and promotional descriptions of adaptive music platforms substantially outrun the peer-reviewed evidence base characterized here.

## 5 Conclusion

Algorithmically personalized music interventions are associated with measurable neurophysiological change, principally in frontal and prefrontal oscillatory activity. Eight studies employed comparators capable of isolating an algorithmic component from music exposure, and all eight favored the algorithmic condition. They address three different causal questions between them, they test different architectures, and no finding among them has been independently replicated, so the signal is preliminary and hypothesis-generating. The evidence base is small and methodologically immature. Most studies cannot distinguish algorithmic personalization from music exposure, none measured expectancy in an intervention whose technological framing invites it, the median reported sample was twenty-two participants, and GRADE certainty is very low for every outcome domain assessed. The architecture that theory most strongly motivates, generative individualized content under real-time closed-loop adaptation, has been demonstrated in a single small emotion-induction study and never against a clinical or health outcome. Neurophysiological change, behavioral change, and clinical benefit remain separate claims, and this literature supports the first far better than the third. Adequately powered trials with yoked-feedback controls, pre-registered analysis plans, measured expectancy, full reporting of the personalization algorithm and of the musical material, and independent replication are required before this class of intervention can be evaluated clinically.
